# Impact of metal salts on the survival, development, and oviposition behavior of coconut rhinoceros beetle (Coleoptera: Scarabaeidae)

**DOI:** 10.3389/finsc.2023.1157769

**Published:** 2023-05-24

**Authors:** Tomie Vowell, Megan E. Manley, Jordie R. Ho, Shizu Watanabe, Michael J. Melzer

**Affiliations:** Agrosecurity Laboratory, Department of Plant and Environmental Protection Sciences, College of Tropical Agriculture and Human Resources, University of Hawai’i, Honolulu, HI, United States

**Keywords:** invasive beetle management, Epsom salt, larval development, biosolids, coconut rhinoceros beetle

## Abstract

*Oryctes rhinoceros* (Coleoptera: Scarabaeidae) is an invasive pest of palms in the Pacific Region, including Hawaii, for which limited management options are available. O. rhinoceros larvae develop in materials rich in organic materials such as green waste and animal manure. Biosolid waste within Hawaii’s infestation zone, however, was determined to inhospitable to *O. rhinoceros*. To determine if the elevated metal salts present in the biosolid waste was responsible for this observation, *O. rhinoceros* life stages were acutely and chronically exposed to several metal salts at increasing concentrations to determine the impact of these salts on survival, development, and oviposition behavior. Acute (7 days) exposure to mulch rehydrated in solutions of CaCl_2_, KCl, MgCl_2_, or NaCl increasing in concentration from 0 to 0.7 M resulted in increased mortality, with concentrations > 0.5 M generally being 100% lethal to both first and second-instar larvae. A similar trend in survival was observed in subsequent experiments using a horticultural grade of Epsom salt (MgSO_4_) at 1×, 2×, and 5× the recommended application rate. Chronic exposure (eggs reared on mulch through pupation) to Epsom salt at these same rates resulted in significantly delayed instar development and reduced adult biometrics at both 1× and 2× levels. Similar to the acute exposure, eggs exposed to 5× levels did not hatch, or the first instar died soon after emergence. In choice experiments, gravid females showed no oviposition preference for media hydrated with water or 2× Epsom salt but did avoid ovipositing in mulch rehydrated in 5× Epsom salt. These trials demonstrate a potentially novel approach to managing pest populations of *O. rhinoceros*.

## 1 Introduction

The coconut rhinoceros beetle [CRB; *Oryctes rhinoceros* L. (Coleoptera: Scarabaeidae)] is a damaging pest of numerous palm species, particularly outside its native range (1, 2). Coconut palm (*Cocos nucifera*) appears to be the preferred host of CRB, although oil (*Elaeis guineensis*), date (*Phoenix dactylifera*), and numerous ornamental palm species are also common hosts (2–4). Adult beetles burrow into palm crowns to feed on sap, causing foliar damage known as “V-cuts” to unfurled fronds. Tree death may occur if the meristem is sufficiently damaged. CRBs oviposit in decomposing plant material or soil with high organic content. The three larval instars feed on this organic material, further breaking it down, and the beetles are not considered damaging during these life stages.

A breeding population of CRBs was discovered in O'ahu, Hawai’i, in 2013 (5), leading to the implementation of a large, multi-agency response and eradication effort. Ongoing delimiting and monitoring surveys using ground and aerial traps provide both metrics of the CRB population and the removal of adults from the environment. In addition, accumulations of green waste or other suitable material for CRB oviposition and larval development are routinely monitored within the current infestation zone and are removed or sanitized when CRB life stages are present. Within the CRB infestation zone on O'ahu are large accumulations of biosolids, the solid end product of wastewater treatment. Although no evidence of CRB life stages was present in these biosolid accumulations, it was deemed essential as part of the response and eradication effort to determine if these materials could support larval development. In this study, we evaluated whether CRB could survive and complete its life cycle in these materials. As an outcome of these experiments, we evaluated the impact of metal salts, with an emphasis on Epsom salt, on the survival, development, and behavior of CRB under laboratory conditions. Our results indicate that the application of common metal salts to breeding site material represents a potentially inexpensive approach for managing CRB that can be incorporated into current efforts.

## 2 Materials and methods

### 2.1 Coconut rhinoceros beetle colony

Beetle eggs, larvae, and adults used in these experiments were drawn from a CRB colony that had been established using adults collected from traps deployed in the field. These field-caught specimens were quarantined for at least 7 days within the Hawai’i Department of Agriculture Plant Quarantine facilities to ensure that they harbored no obvious pests or pathogens then transferred to the University of Hawai’i Manoa Arthropod Containment Laboratory (UH-ACL). Adult beetles collected from the field (founders) were placed in boxes (26 cm × 19 cm × 17 cm) containing a mulch mixture and allowed to mate and lay eggs.

Initially, a 50:50 (v/v) mixture of the commercial compost Menehune MAGIC™ (Hawaiian Earth Products, Kapolei, HI, USA) and coconut coir peat (Pacific Grower Supplies, Hilo, HI, USA) was used as the medium for all life stages and the food source for larvae. In 2017, the mulch used in the colony transitioned to green waste that is pruned from campus plants and processed by the University of Hawaii's Landscaping Services. The Landscape Services mulch was sieved through either a 1.6-mm or a 6.4-mm mesh to remove large pieces of material, creating either “fine” or “coarse” mulch, respectively. The coarse mulch was used in the adult boxes.

The F1 generation was reared to the adult stage. As eggs were collected from the founder boxes with adults, they were placed in individual containers (26 cm × 19 cm × 17 cm) containing mulch. The larvae then fed upon the mulch, pupated, and emerged as adults, which were fed as described below. This new generation of CRB adults was allowed to mate. After the successful production of an F2 generation, adults of the founder, F1, and F2 generations were commingled. Up to 15 adult CRBs with a female-to-male ratio of approximately 2:1 were housed in plastic containers (43 cm × 28 cm × 17 cm) containing coarse mulch. Eggs were collected weekly and transferred to plastic containers containing fine mulch, up to 300 eggs/container. After reaching the late second or early third instar, larvae were transferred to individual 1-L bottles containing coarse mulch until pupation and imago emergence. Rearing took place in I-41LLVL Biological Incubators (Percival Scientific, Perry, IA, USA) at temperatures between 28°C and 30°C, and the medium was exchanged as needed. Humidity was provided by placing buckets of water within the incubator. Adult beetles were fed a solid artificial diet once per week, amounting to approximately 13 g/beetle/week. The diet consisted of 33 g of sugar and 26 g of Muscle Milk vanilla-flavored 100% Whey Protein Isolate and Concentrate (Cytosport, Walnut Creek, CA, USA) dissolved in 1 L of boiling water, and solidified with 15 g of agar (Fisher BioReagents, Pittsburgh, PA, USA). All specimens used in this study came from offspring produced by the commingled founder, F1, and F2 adult beetles, and all experimentation was conducted within the UH-ACL.

### 2.2 Biosolid analysis

Approximately 500 g of biosolids collected from a single location within the CRB infestation zone was thoroughly examined to ensure that no CRB life stages were present. These biosolids were then submitted to the University of Hawai’i Agriculture Diagnostic Service Center to be analyzed for pH, electroconductivity (EC), and concentration of phosphorus (P), potassium (K), calcium (Ca), magnesium (Mg), and sodium (Na) (6). The resulting values were compared with the range of values for soils in Hawai’i (7, 8).

### 2.3 Larval survival

To evaluate the ability of CRBs to survive and colonize biosolids, eggs collected from the colony the day prior were placed individually in 111-cc translucent plastic containers with a plastic lid with four holes pierced in the top for air exchange (Dart Container Corporation, Mason, MI, USA). Each cup contained 100% biosolids, a mixture of fine mulch and biosolids (75%, 50%, or 25% biosolids, v/v), or 100% fine mulch (control treatment), as described for colony rearing. Each container held approximately 100 cc of one of the biosolid mixtures, and a single egg was placed on the surface and lightly covered with the mulch.

Ten eggs were evaluated for each of the four mixtures and the control. Eggs were initially checked after 2 weeks, and weekly thereafter, for hatching. If the egg hatched, the larva was monitored weekly for its mortality, instar stage, and weight. After 5 weeks, the surviving larvae were transferred to larger (≈ 575 cc) Magenta™ GA-7 vessels (Sigma-Aldrich, St. Lois, MO, USA) with perforated lids, containing freshly made mulch at the same biosolids to fine mulch ratio. Monitoring for larval survival, instar, and weight continued until all surviving larvae for the control treatment reached the third instar. If a larva was dead, it was noted as “dead” and no further records were maintained for that individual. Unhatched eggs were returned to the container for continued observation until the egg hatched or the egg was not found for 2 consecutive weeks.

If an egg or larva was not found, mulch was kept for another week of observation in case the egg or first instar was missed. If nothing was found for 2 consecutive weeks of observation, that egg/larvae was determined to be “dead”. If larvae were found alive after 1 week of missed observation, the life stage and weight were recorded and monitoring continued. If a larva was found after 1 week of missed observation and it was dead, it was noted as “dead” and no further records were maintained for that individual. The procedure was replicated three times.

Survival curves were created and plotted for overall larval survival in each media treatment using the Surv function from the Survival package (9) in R (10). Time was reported in the number of weeks from when eggs entered each medium until larval death or the end of the observation period. A pairwise comparison of survival curves by log-rank tests was used to determine the specific significance between treatments.

### 2.4 Acute exposure to metal salts

To evaluate the survival of CRBs in mulch containing incremental concentrations of common metal salts, the Menehune MAGIC™ potting mix was spread out and dehydrated in aluminum pans for at least 1 day in an oven at 120°C. Single preliminary trials were conducted with salt solutions at 0.25 M, 0.5 M, 0.75 M, and 1 M concentrations. Deionized water (dH_2_O) served as a control treatment. Each cubic centimeter of dried mulch was rehydrated with 0.46 mL of salt solution or dH_2_O and placed in a Magenta™ GA-7 vessel or 32-oz round culture vessel (*Phyto*Technology Laboratories, Lenexa, KS, USA) with a perforated lid. Sodium chloride (NaCl, m.w. 58.44), potassium chloride (KCl, m.w. 74.55), magnesium sulfate (MgSO_4_, m.w. 120.37), calcium sulfate (CaSO_4_·2H_2_O, m.w. 172.17), magnesium chloride (MgCl_2_·6H_2_O, m.w. 203.3), and calcium chloride (CaCl_2_·2H_2_O, m.w. 147.02) were the metal salts selected for evaluation. To evaluate these treatments, first-instar larvae and second-instar larvae were placed in separate communal containers and incubated at 30°C. Each treatment had five of each instar stages. Larvae were evaluated after 1 week for survival. Those larvae that disintegrated and whose remains were not recovered were considered dead. Three replicates were performed for each instar stage and salt concentration.

The results from the preliminary trials were used to predict a range of concentrations of the various salts that might be lethal to the first and second instars of CRBs. These subsequent trials followed the same methodology as the preliminary trials, but the range of concentrations of salts (in 0.1M increments) varied depending on the larval stage and salt used. After the initial test with MgSO_4_, subsequent tests were conducted using a commercially available Epsom salt (MgSO_4_·7H_2_O; Gro Tec, Inc., Madison, GA, USA) based on the application rate on the package. Treatments of 1× (0.13 M), 2× (0.26 M), and 5× (0.64 M) of the recommended application rate of Epsom salt was used to test both the first and second instars.

The survival data for the secondary trials were analyzed using R software (10) with the dplyr package (11). Univariate ANOVAs were conducted for each life stage (first or second instar) and for each salt (sodium chloride, potassium chloride, Epsom salt/magnesium sulfate, calcium sulfate, magnesium chloride, and calcium chloride) tested. Analyses of instar stages across salt treatments were not combined because different salt concentrations were used for the first and second instars. For each ANOVA, a Tukey honestly significant difference (HSD) test was conducted to determine differences in larval (first or second instar) survival at different concentrations of the salt tested.

### 2.5 Acute and chronic exposure to Epsom salt

The effects of acute and chronic exposure of CRB first and second instars to a commercial formulation of Epsom salt (MgSO_4_·7H_2_O) intended for horticultural applications (Gro Tec, Inc.) were evaluated. Replicated acute exposure trials were conducted as described above, except that the Menehune MAGIC™ potting mix was replaced with the fine mulch sourced from UH landscaping, as used in the colony maintenance. The mulch was rehydrated using Epsom salt concentrations at 1×, 2×, and 5× the manufacturer’s recommended application rate for potted palms (0.5 cup/gallon of water), which was estimated to be 0.13, 0.26, and 0.64 M, respectively. The control treatment consisted of rehydration with dH_2_O. For each treatment, the percentage that survived to each larval stage was analyzed by ANOVA and the Tukey HSD test, as described above.

For chronic exposure, control and Epsom salt-treated fine mulch was placed into the 111-cc translucent plastic containers described above. An individual CRB egg was placed in the center of each container and covered with approximately 5 mm of fine mulch, as described for colony rearing. The containers were maintained at 30°C and weekly observations began after 2 weeks. If larvae were present, their life stage and weight were recorded, then the larvae were returned to their containers. If the eggs were still present and appeared vital, they were placed back into the cup for further observation. If the eggs were missing or in poor condition, or there was no evidence of larvae following 2 weeks of observations, the egg was considered dead and no further records were kept for that specimen. All cups with samples were returned to 30°C and checked weekly for life stage and weight. After 5 weeks, the mulch was replaced with fresh mulch. After 10 weeks, larvae were moved into Magenta™ GA-7 vessels with fresh mulch to accommodate larval growth. Mulch was replaced after 13 weeks, and again after 19 weeks if there was no evidence of pupation. Once pupation was initiated, weekly measurements ceased, due to the fragility of this life stage, but the containers were monitored for emergence. Once adults emerged, measurements of beetle length, elytron length, width, height, and weight were recorded. Three independent replicates were performed concurrently, with 10 eggs per treatment for each replicate.

As described for the biosolid trials, survival curves were created and plotted for overall larval survival. Curves were created for each using the Surv function from the Survival package (9) in R (10). Time was reported in the number of weeks from when eggs entered each medium until larval death or the end of the observation period. A pairwise comparison of survival curves by log-rank tests was used to determine the specific significance between treatments.

### 2.6 Epsom salt and oviposition behavior

To determine if the presence of Epsom salt had an impact on the oviposition behavior of female CRBs, choice tests were conducted with specimens caught from the environment and maintained in the colony at the UH-ACL. These tests were performed under laboratory conditions in a wooden chamber with an arena composed of three compartments. One compartment contained sand and served as a putatively undesirable substrate for oviposition. The other two compartments contained green waste that had been passed through a 13-mm screen. The green waste in one compartment was dehydrated as described above and rehydrated with dH_2_O. The other compartment contained green waste that was rehydrated with either 2× or 5× Epsom salt solution. The contents of all three compartments were flush with the dividers, with no surface obstacles between treatments. A single female was placed in the sand compartment, the lid of the chamber was closed and secured, and the chamber was stored at 30°C. The experiment concluded after 1 week when the compartments were excavated, and the number of eggs recovered from each compartment was recorded. A different female and fresh mulch and sand were added to each compartment at the beginning of each replication. A total of 12 replicates were performed for each Epsom salt concentration (2× and 5×).

For each choice experiment, the number of eggs laid per beetle in each substrate was compared using a non-parametric Wilcoxon rank-sum test (JMP Pro 13). If no eggs were found in any substrate, this category was not included in the analysis. The oviposition pattern for each beetle was scored and analyzed using categorical data analysis techniques. A single female beetle was scored as laying her eggs in mulch with Epsom salt added, mulch without salt added, sand, both mulches, or none. The null hypothesis, that oviposition would be equally distributed across substrates, was tested using a chi-squared goodness-of-fit test (JMP Pro 13). Where appropriate, a subdivided chi-squared goodness-of-fit test (12) was subsequently conducted to determine if the distribution of ovipositing was the same in both mulch substrates within the same trial, or if one of the two mulch substrate options was preferred. The sand category was not included in any subsequent analysis, as no beetles were ever found to oviposit in that substrate.

## 3 Results

### 3.1 Biosolid analysis

An analysis of the biosolid material within the CRB infestation zone and comparison with the expected values for heavy agricultural soils in Hawai’i revealed that the biosolid pH, P, and Ca values were similar to those expected, whereas the EC, K, Mg, and Na values were very highly elevated (**Table 1**). These elevated values suggest that the biosolid material would not support the growth of most agricultural crops (7).

**Table 1:** Chemical analysis of the biosolid materials used in this study compared with the expected range for heavy soil in Hawai’i.

Component (unit)	Biosolid	Expected (heavy soil)	Biosolid designation
pH	5.9	5.8–6.2	Normal
Electrical conductivity (mmhos/cm)	23	< 3.0	Very high
Phosphorus (ppm)	25	25–35	Normal
Potassium (ppm)	1579	200–300	Very high
Calcium (ppm)	2158	1,500–2,000	High
Magnesium (ppm)	1417	300–400	Very high
Sodium (ppm)	1508	81–120	Very high

### 3.2 Larval survival

**Figure 1:**
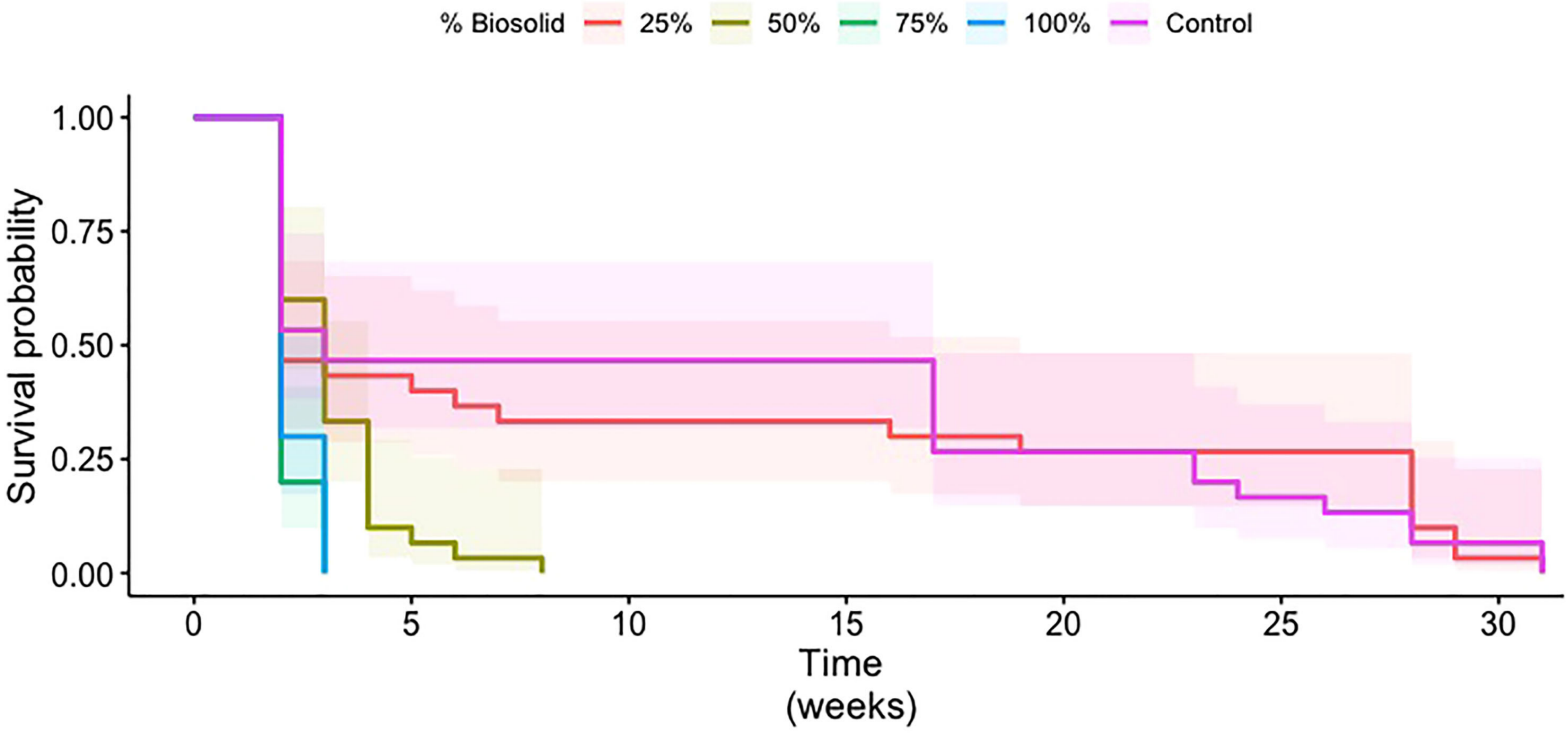
Survival probability curves of coconut rhinoceros beetle larvae in media containing 0% (control), 25%, 50%, 75%, or 100% biosolid materials. Time 0 is when eggs were deposited in the different media treatments. The shaded areas represent 95% confidence intervals for each treatment.

Treatments with 25% biosolids had higher survival probabilities than treatments with 50% or more biosolids (**Figure 1**). Survival probabilities for treatments with 25% biosolids appeared comparable to survival probability with the control treatment. In addition, survival probability was similar for the 75% and 100% biosolid treatments. The pairwise comparison of the survival curves confirmed that there was no significant difference in the survival of larvae between the 25% biosolid medium and the control (*p* = 0.97). Additionally, the survival probabilities did not differ significantly between the 75% and 100% biosolid treatments (*p* = 0.42). Larval survival in the treatment with 50% biosolids was significantly different from survival with the control treatment (*p* = 2.31e–3) and from survival with the 25% (*p* = 1.159e–2), 75% (*p* = 4.4e–4), and 100% (*p* = 1.17e–3) biosolid treatments.

### 3.3 Acute exposure to metal salts

**Figure 2:**
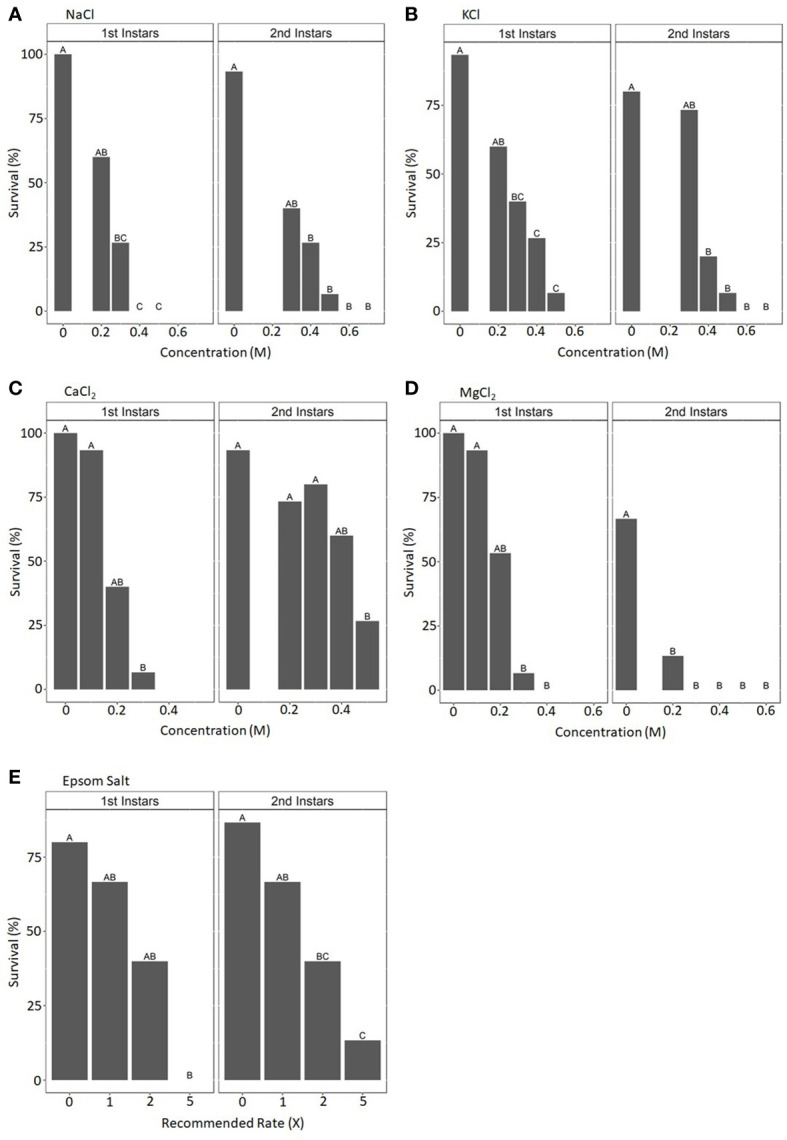
Mean percentage survival rates for coconut rhinoceros beetle first- and second-instar larvae after 1 week’s exposure to mulch saturated with solutions containing various concentrations of NaCl **(A)**, KCl **(B)**, CaCl_2_ **(C)**, MgCl_2_ **(D)**, or Epsom salt **(E)**. Mean values represent three replicates of the survival of five larvae in each condition. Treatment concentrations within the salt type and larvae stage not sharing the same letter are significantly different (NaCl: first instars, *F*_4,8 _= 16.857, *p* < 0.001; second instars, *F*_5,10 _= 15.518, *p* < 0.001; KCl: first instars, *F*_4,8 _= 5.041, *p* < 0.05; second instars *F_5_*_,10 _= 17.056, *p* < 0.001; CaCl_2_: first instars, *F*_3,6 _= 12.182, *p* < 0.01; second instars, *F*_4,8 _= 11.15, *p* < 0.01; MgCl_2_: first instars, *F*_4,8 _= 14.5, *p* < 0.001; second instars, *F*_5,8 _= 10.667, *p* < 0.001; Epsom salt: first instars, *F_3,6 _=* 6.588, *p* < 0.05; second instars, *F*_3,6 _= 11.957, *p* < 0.01).

The survival of first- and second-instar CRB larvae was evaluated after 7 days of exposure to mulch containing incremental concentrations of six different salts: NaCl, KCl, MgSO4, CaSO_4_·2H_2_O, MgCl_2_·6H_2_O, and CaCl_2_·2H_2_O. All salts significantly reduced the survival of these larvae as their concentrations in the mulch increased (**Figures 2A–D** and data not shown). Survival rates were typically higher for second-instar larvae than for first-instar larvae. Mulch rehydrated with solutions greater than 0.5 M in concentration, regardless of the salt used, typically resulted in no survival of either first- or second-instar larvae after 7 days of exposure.

### 3.4 Acute and chronic exposure to Epsom salt

**Figure 3:**
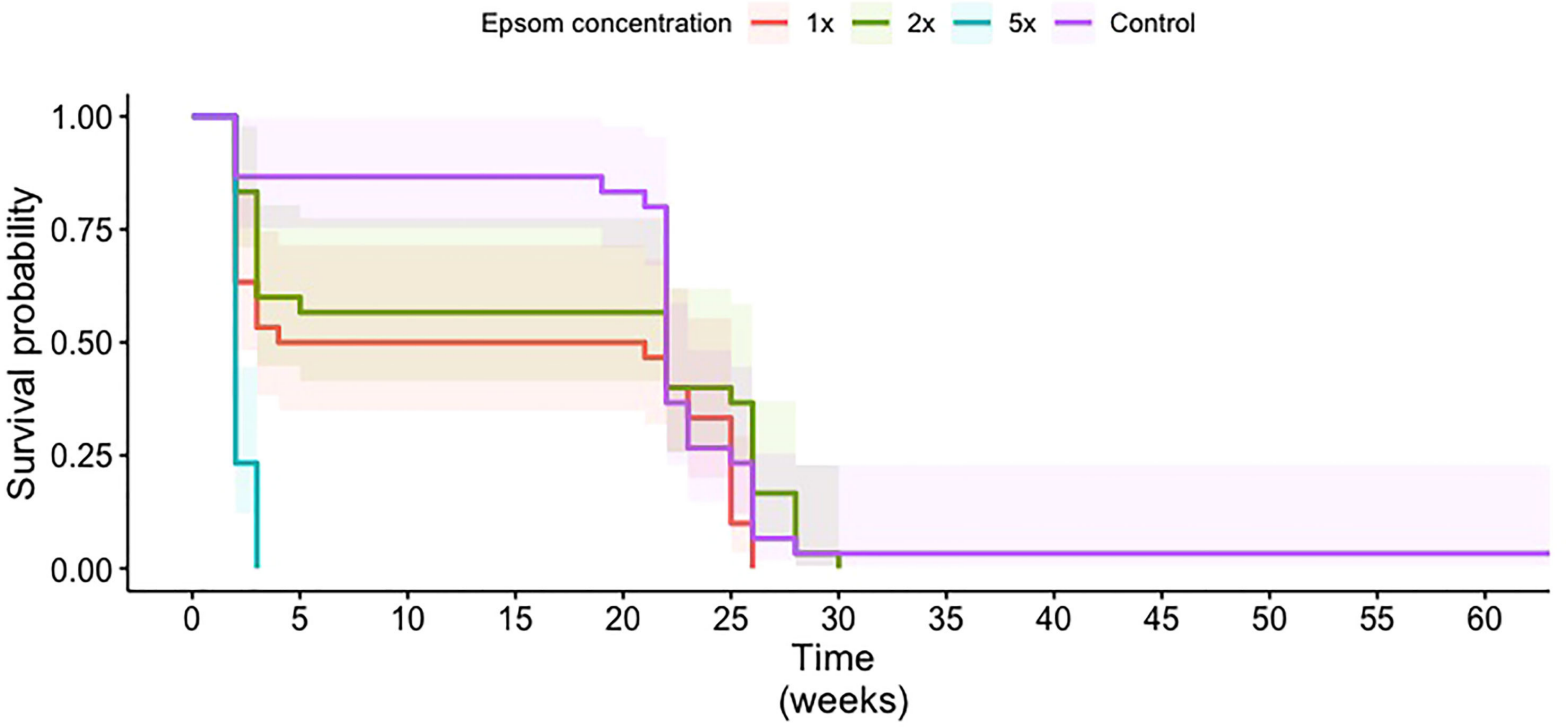
Survival probability curves of coconut rhinoceros beetle larvae in media treated with water (control) or Epsom salt at 1×, 2×, or 5× of the manufacturer’s recommended rate for palms. Time is in weeks and starts when the egg entered the medium. Shaded areas represent 95% confidence intervals for each treatment.

A commercially available Epsom salt intended for horticultural applications was evaluated for its impact on CRB survival following acute exposure. At 1×, 2×, and 5× the recommended application rate following a 7-day (acute) exposure, the survival of first- and second-instar larvae decreased as the amount of Epsom salt in the mulch increased, with no first-instar larvae surviving the 5× treatment (**Figure 2E**). Following chronic exposure, overall larvae survival probabilities were higher for the control, 1×, and 2× treatments than for the 5× treatment. They also did not appear to be significantly different from each other (**Figure 3**). This was confirmed with the pairwise log-rank test. The 5× treatment was significantly different from all other treatments (control: *p* = 3.0e–11; 1×: *p* = 8.2e–6; 2×: *p* = 4.0e–8). The only other significant difference between treatments was between the 1× and 2× treatments (*p* = 0.049).

**Figure 4:**
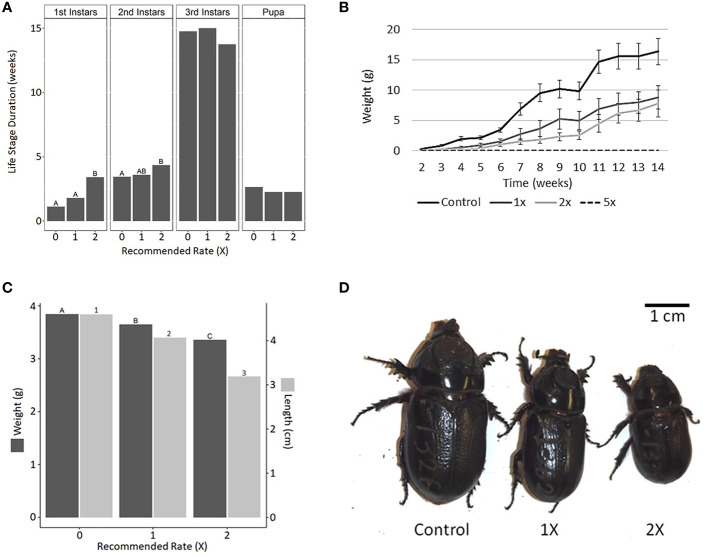
Effects of Epsom salt on the growth and development of coconut rhinoceros beetle (CRB). Epsom salt was added to the rearing mulch at 1×, 2×, and 5× the manufacturer’s recommended rate for palms and the development of CRB was monitored from egg to adult. **(A)** The addition of Epsom salt significantly delayed the development of larvae into second and third instars but did not significantly impact the duration of the third-instar or pupal phase. **(B)** Larval weight gain was reduced with increasing concentration of Epsom salt in the mulch. **(C)** Adult biometrics were negatively impacted by the presence of Epsom salt during larval development, resulting in noticeably smaller adult beetles **(D)**. Epsom salt at the 5× rate was lethal to CRB larvae, and therefore the results of this treatment are not shown in any parts of this figure. Data with different letters or numbers are significantly different (life stage duration: first instar, *F*_2,52 _= 11.39, *p* < 0.001; second instar, *F*_2,52 _= 4.16, *p* < 0.05; third instar, *F*_2,51 _= 1.114, *p* = 0.336; pupa, *F*_2,50 _= 2.91, *p* = 0.122; adult biometrics: weight, *F*_2,50 _= 23.434, *p* < 0.001; length, *F*_2,50 _= 21.839, *p* < 0.001).

The development of first- and second-instar CRB larvae (but not third-instar larvae or pupae) was delayed when chronically exposed to mulch containing 2× the recommended rate of Epsom salt (**Figure 4A**). Furthermore, the average weight of the larvae decreased with increasing levels of Epsom salt in the mulch (**Figure 4B**). The body weight and length of newly emerged adult CRBs were also reduced with increasing Epsom salt levels (**Figures 4C, D**). Similar to acute exposure experiments, no larvae survived in mulch supplemented with 5× the recommended rate of Epsom salt.

### 3.5 Oviposition choice tests

Female CRBs laid on average ( ± SE) 29 ± 9 eggs when presented with the choices of mulch amended with 2× Epsom salt, unamended mulch, and sand. Although slightly more eggs were laid by larvae receiving the 2× Epsom salt treatment than by larvae subjected to the control treatment, the difference was not statistically different (**Table 2**). No beetles laid eggs in the sand. Three of the 12 beetles oviposited in both 2× Epsom salt and control treatments, eight chose either the 2× Epsom salt (*n* = 3) or control (*n* = 5) treatments, and one beetle did not oviposit. The numbers of females that laid eggs in either the 2× Epsom salt or control treatment, or both, within the same trial were equally distributed (χ^2 ^= 0.73, df = 2, *p* = 0.6951), and there was no significant preference for the 2× Epsom salt or control treatment (χ^2 ^= 0.50, df = 1, *p* = 0.4795).

**Table 2:** The mean number of eggs (± SE) laid and mean percent of total eggs laid by coconut rhinoceros beetle when provided with a choice of unadulterated oviposition mulch (control) or oviposition mulch treated with Epsom salt at twice the recommended rate as a soil amendment (2× Epsom salt).

Substrate	Eggs	Percent of eggs
Control	12 ( ± 7)	42
X× Epsom salt	17 ( ± 6)	49
	*t* = 0.1546, df = 11, *p* = 0.8800	

Female CRBs laid on average ( ± SE) 38 ± 6 eggs when presented with the choice of 5× Epsom salt-amended mulch, unamended mulch, or sand. Significantly more eggs were laid in the control treatment than in the 5× Epsom salt treatment (**Table 3**). No beetles laid eggs in the sand. Three of the 12 beetles oviposited in both the 5× Epsom and control mulch, one oviposited in the 5× Epsom salt treatment, seven oviposited in the control mulch, and one did not oviposit. The numbers of females that laid eggs in either the 5× Epsom salt or control treatment, or both, or neither, within the same trial were not equally distributed (χ^2 ^= 8.00, df = 3, *p* = 0.0460), and there was a significant preference for the control treatment over the 5× Epsom salt treatment and sand (χ^2 ^= 4.50, df = 1, *p* = 0.0339).

**Table 3:** The mean number of eggs (± SE) laid and mean percent of total eggs laid by coconut rhinoceros beetle when provided with a choice of unadulterated oviposition mulch (Control) or oviposition mulch treated with Epsom salt at 5× the recommended rate as a soil amendment (5× Epsom salt).

Substrate	Eggs	Percent of eggs
Control	33 ( ± 7)	75
5× Epsom salt	5 ( ± 3)	17
	*t* = 3.1380, df = 11, *p* = 0.0094	

## 4 Discussion

In this study, we evaluated the impact of metal salts, with an emphasis on Epsom salt, on the survival, development, and behavior of CRBs under laboratory conditions. All salts evaluated appeared to have lethal and sublethal impacts on CRB, depending on life stage and salt concentration. In general, mulch rehydrated with salts at concentrations higher than 0.4–0.5 M resulted in 100% mortality for early CRB instars after short-term exposure (as a reference, the typical molarity of seawater is approximately 0.6 M). Because many metal salts, such as NaCl and MgCl_2_, have negative impacts on plant germination and microbial decomposition when present in soils at elevated levels (13–15) we focused on Epsom salt, which is a common soil amendment and fertilizer (16). In addition to elevated mortality following acute exposure at 1×, 2×, and 5× the recommended rate, chronic exposure led to significant developmental delays and reduced larval and adult size. Although elevated mortality is the desired outcome from a management perspective, the developmental delays and reduced larval and adult size may also be beneficial to Hawai’i’s eradication program, based on our observations with the CRB colony at the UH-ACL. The first instar of CRB experiences the highest mortality rate and appears to be most susceptible to environmental stresses as well as putative parasites, predators, and pathogens. This elevated mortality was observed in several of the control treatments involving first instars in this study (**Table 2**, **Figure 2E**). Prolonging this life stage may result in even higher mortality, and the incorporation of Epsom salt should be a consideration for future larval bioassays evaluating entomopathogens. We have observed that diminutive adult CRBs typically have shortened lifespans in the UH-ACL colony, particularly when they are integrated into rearing boxes with adults of typical size. Often these smaller individuals are decapitated within days of integration. It is unclear what type of adult–adult interaction is responsible for this observation. Furthermore, small adult females display reduced fecundity (17). Therefore, it is plausible that small adult CRBs will have reduced fitness in the environment, which is highly desirable for management or eradication programs.

We evaluated whether female CRBs would oviposit in mulch amended with Epsom salt under laboratory conditions. Our observations indicated that ovipositing females were not repelled by Epsom salt at the 2× treatment but did display repulsion to the 5× treatment. If the impact of Epsom salt observed in the laboratory successfully translates into an approach for CRB management in the field, its use may be dictated by the response scenario. In Hawai’i, the incipient CRB population is highly localized and subject to eradication. Therefore, tools must be used in a manner that minimizes the potential for spread to uninfested areas. Hypothetically, Epsom salt, if used to treat oviposition sites, should be administered at rates that do not discourage oviposition, as this repulsion may result in the further dispersal of gravid females in search of new locations to oviposit. Conversely, in locations where CRB populations are widespread and the goal is to reduce the population, such as an oil palm plantation, Epsom salt should, hypothetically, be used at higher rates that both sanitize oviposition sites and discourage oviposition.

The use of metal salts appears to be a largely unexplored approach for managing invasive and pest insects. Gaugler (18) characterized the negative impact sodium chloride had on the survival and development of the stable fly (*Stomoxys calcitrans*) under laboratory conditions and suggested the nearshore application of seawater as an alternative to the applications of methoxychlor. The use of unspecified or common salt either alone or in combination with sand and ash has also been reported as a field management tool for CRB (19, 20), which coincidentally is also the target organism of this study. Anithakumari et al. (20) did not evaluate the use of salt but rather reported it as a tool used by the community for CRB management. In this approach, salt, combined with sand and ash, was applied to the leaf axils of coconut palms and perhaps acted as a deterrent to feeding by adult CRB. Varma (19) did evaluate common salt, although the site of application was not defined. In this approach, common salt was applied at 2 kg/palm twice a year, resulting in a 23% reduction in larvae in comparison with the control treatment.

Epsom salt is relatively inexpensive and is commonly used as a management tool for snails and slugs in home gardens, and magnesium sulfate (specifically the Mg ion) is also toxic to the freshwater snail, *Amerianna cumingi*, particularly when calcium is limited (21). However, little information is available for broader pest management use. The results of our study warrant field evaluation of Epsom salt as a management tool for CRB larvae and perhaps the larvae of other subterranean insect pests. Such evaluations could address efficacy and cost in comparison with established management tools but must also consider phytotoxicity and the potential environmental impact of this approach.

## Data availability statement

The original contributions presented in the study are included in the article/supplementary materials. Further inquiries can be directed to the corresponding author.

## Author contributions

MJM, SW, and TV contributed to the conception and design of the study. TV, MEM, and SW performed the experiments. TV and JH performed the statistical analysis. MJM drafted the manuscript, with TV, JH, and MEM writing sections of the manuscript. All authors contributed to the article and approved the submitted version.
